# Bronchoperitoneal fistula in a patient with pneumatosis Cystoides intestinalis: a diagnostic challenge

**DOI:** 10.1093/omcr/omag116

**Published:** 2026-07-08

**Authors:** Idan Grossmann, Aubin Attila, Anid Hassan, Anasuya Brahmamdam, Jaimin Dudhia, William Sebastian, Stuart Campbell, Aroob Farooqi, Kamil Ahmad Kamil, Mohammad Amir Hossain, Lee Peng

**Affiliations:** Department of Internal Medicine, Hackensack Meridian Health Jersey Shore University Medical Center, 1945 NJ-33, Neptune City, NJ, 07753, United States; Department of Internal Medicine, Hackensack Meridian Health Jersey Shore University Medical Center, 1945 NJ-33, Neptune City, NJ, 07753, United States; Department of Internal Medicine, Hackensack Meridian Health Jersey Shore University Medical Center, 1945 NJ-33, Neptune City, NJ, 07753, United States; Hospitalist, Hackensack Meridian Health Jersey Shore University Medical Center, 1945 NJ-33, Neptune City, NJ, 07753, United States; Hospitalist, Hackensack Meridian Health Jersey Shore University Medical Center, 1945 NJ-33, Neptune City, NJ, 07753, United States; Department of Surgery, Hackensack Meridian Health Jersey Shore University Medical Center, 1945 NJ-33, Neptune City, NJ, 07753, United States; Department of Thoracic Surgery, Hackensack Meridian Health Jersey Shore University Medical Center, 1945 NJ-33, Neptune City, NJ, 07753, United States; Department of Medicine, Postgraduate Medical Institute, Ameer-ud-Din medical college, 6 Birdwood Road, Jinnah Town, Lahore, 54000, Pakistan; Department of Internal Medicine, Mirwais Regional Hospital, Nawy Sarak near to Shahidano Chowk, District 1, 3801, Kandahar, Afghanistan; Department of Internal Medicine, Hackensack Meridian Health Jersey Shore University, 1945 NJ-33, Neptune City, NJ, 07753, United States; Department of Gastroenterology, Hackensack Meridian Health Jersey Shore University, 1945 NJ-33, Neptune City, NJ, 07753, United States

**Keywords:** pneumatosis Cystoides intestinalis, Bronchoperitoneal fistula, pneumoperitoneum, non-surgical abdomen, hiatal hernia repair, ventilation-perfusion scan

## Abstract

**Background:**

Pneumatosis cystoides intestinalis (PCI) is a rare condition characterized by the presence of multiple gas-filled cysts within the wall of the gastrointestinal tract, leading to variable clinical presentations and diagnostic challenges. A bronchoperitoneal fistula is an exceedingly rare complication, typically resulting from downward gas tracking secondary to thoracic pathology.

**Case Presentation:**

We report the case of a 76-year-old female with a remote history of hiatal hernia repair who developed chronic gastrointestinal symptoms associated with pneumoperitoneum and PCI, complicated by a bronchoperitoneal fistula. While the most bronchoperitoneal fistulas reported are due to thoracic pathology, we suggest that in this case, the fistula may be associated with PCI-related pneumoperitoneum.

**Conclusions:**

The case demonstrates unique clinical and diagnostic features and raises the possibility of an atypical pathophysiologic association for broncho-peritoneal fistula formation.

## Introduction

PCI is an uncommon clinical condition whose hallmark is the presence of multiple gas-filled cysts within the submucosal or subserosal layers of the intestinal wall. The prevalence of PCI ranges from 0.03% to 0.2%. PCI may be classified as either secondary (85%) or idiopathic (15%), and the clinical spectrum varies from asymptomatic incidental findings to potentially fatal complications [1].

Bronchoperitoneal fistula is an exceedingly rare clinical entity [2]. In most previously reported cases, its development has been secondary to underlying thoracic pathology of varied etiologies. Several cases have been attributed to infectious processes, such as lung abscesses arising from different causes, which subsequently led to fistula formation [3, 4]. Additionally, extension of an infected pancreatic pseudocyst through the diaphragm has been reported as a causative mechanism [5]. Enterobronchial fistulas have also been described in the context of complicated Crohn’s disease [6]. Another important category of etiologies includes postoperative complications, which have been implicated in fistula formation as well [7].

Bronchoperitoneal fistula may be associated with serious complications that can result in life-threatening conditions. Pulmonary complications are common and include recurrent or severe pneumonia [6], as well as pneumothorax [8]. Another major complication is sepsis, which may occur as a result of translocation of enteric pathogens through the fistulous tract [3].

This report describes a 76-year-old patient with chronic non-specific gastrointestinal symptoms who was found to have non-perforated pneumoperitoneum complicated with a bronchoperitoneal fistula.

## Case report

A 76-year-old female with a past medical history of hypertension, type 2 diabetes mellitus, heart failure with reduced ejection fraction (HFrEF), sick sinus syndrome status post pacemaker implantation, and hiatal hernia repair five years earlier, presented with new-onset hematuria. She reported noticing blood on tissue paper after urination but denied abdominal pain, nausea, vomiting, or respiratory symptoms.

The patient also reported a one-year history of progressively worsening intermittent fecal incontinence, characterized by loose, watery, brown bowel movements with excessive flatulence, and an associated unintentional weight loss of 50 pounds during this period.

Review of operative records revealed a laparoscopic hiatal hernia repair without documented intraoperative complications, mesh placement, or postoperative pulmonary sequelae.

Three months earlier, she had been hospitalized for a pacemaker implantation and was incidentally found to have a pneumoperitoneum without any associated symptoms. Subsequently, an upper endoscopy performed during that admission demonstrated normal esophageal, gastric, and duodenal mucosa. No biopsies were obtained. A diagnostic laparotomy was without evidence of bowel perforation.

On the current admission, the patient was alert and hemodynamically stable. The physical examination revealed a soft, non-tender, mildly distended abdomen without rigidity or guarding.

Laboratory results showed a creatinine level of 1.19 mg/dl from a normal baseline.

A computed tomography (CT) of the abdomen and pelvis obtained to evaluate hematuria revealed worsening pneumoperitoneum (Fig. 1) with PCI with multiple air-filled cystic structures within the submucosal layer of the bowel wall (Fig. 2). No definitive etiology for hematuria was identified on imaging, and it was considered an incidental finding unrelated to gastrointestinal presentation. CT angiogram of the chest was unremarkable.

**Figure 1:**
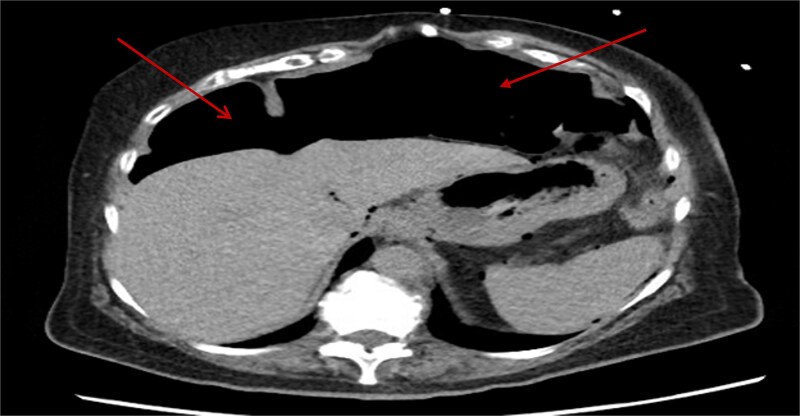
Axial CT of the abdomen demonstrating diffuse pneumoperitoneum.

**Figure 2:**
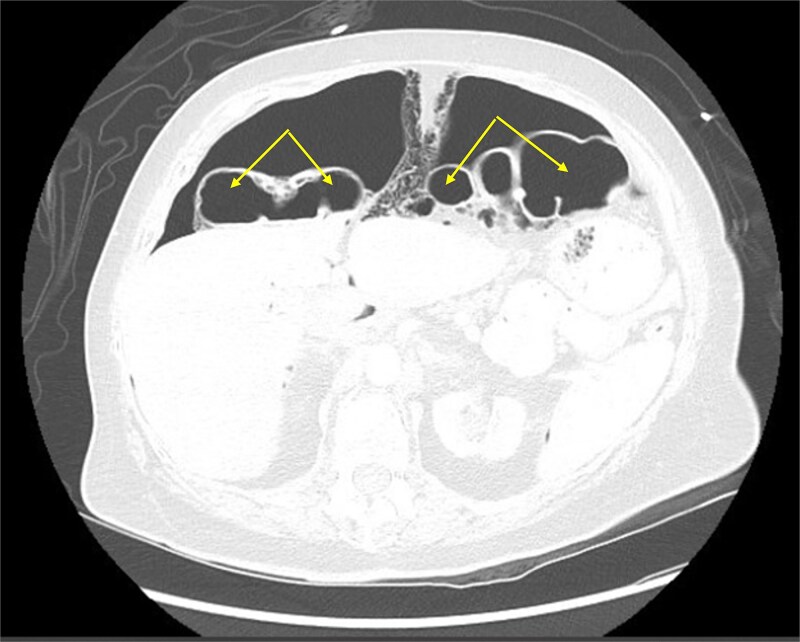
Axial CT of the abdomen image showing multiple submucosal air-filled cysts along the bowel wall, consistent with pneumatosis cystoides intestinalis.

A prior operative report from a previous hospitalization noted a halothane-like odor during the laparotomy, raising suspicion for a fistula. Thoracic surgery team recommended a ventilation-perfusion (V/Q) scan, which revealed findings suggestive of a broncho-peritoneal fistula (Fig. 3 and 4). During the ventilation phase, inhaled radiotracer is normally confined to the lungs; however, delayed imaging exposed tracer accumulation within the abdominal cavity. This finding indicated transdiaphragmatic passage of inhaled gas, providing functional evidence of a broncho-peritoneal fistula. The patient was stable and discharged for outpatient follow-up for fistula repair.

**Figure 3:**
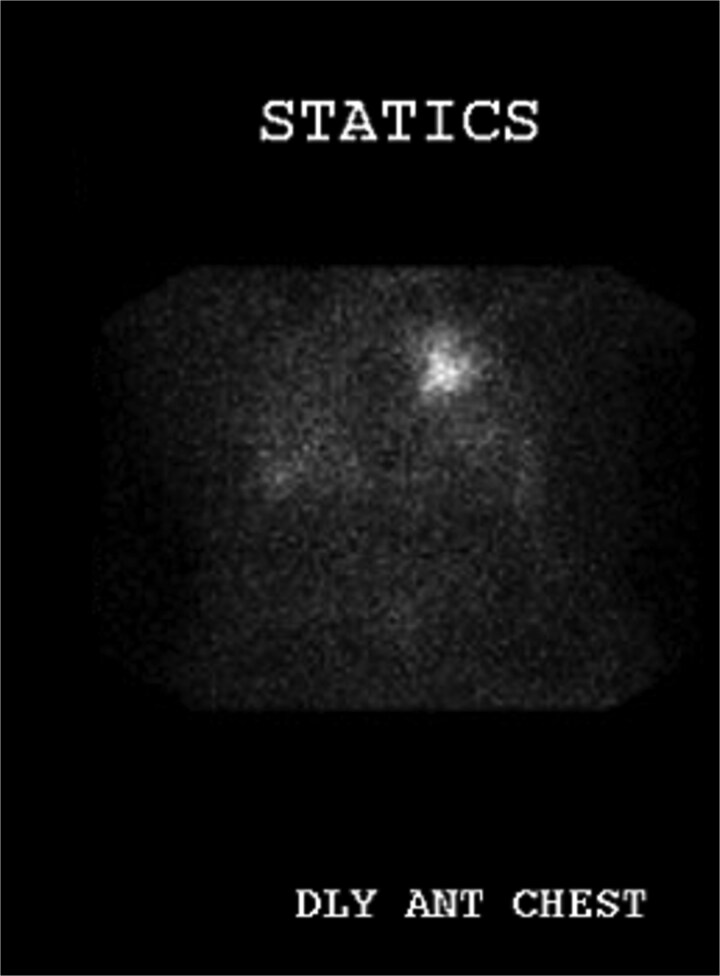
V/Q scan (static image) showing air trapping in the right upper lung and posterior lung bases, suggesting a possible connection to the abdominal cavity.

**Figure 4:**
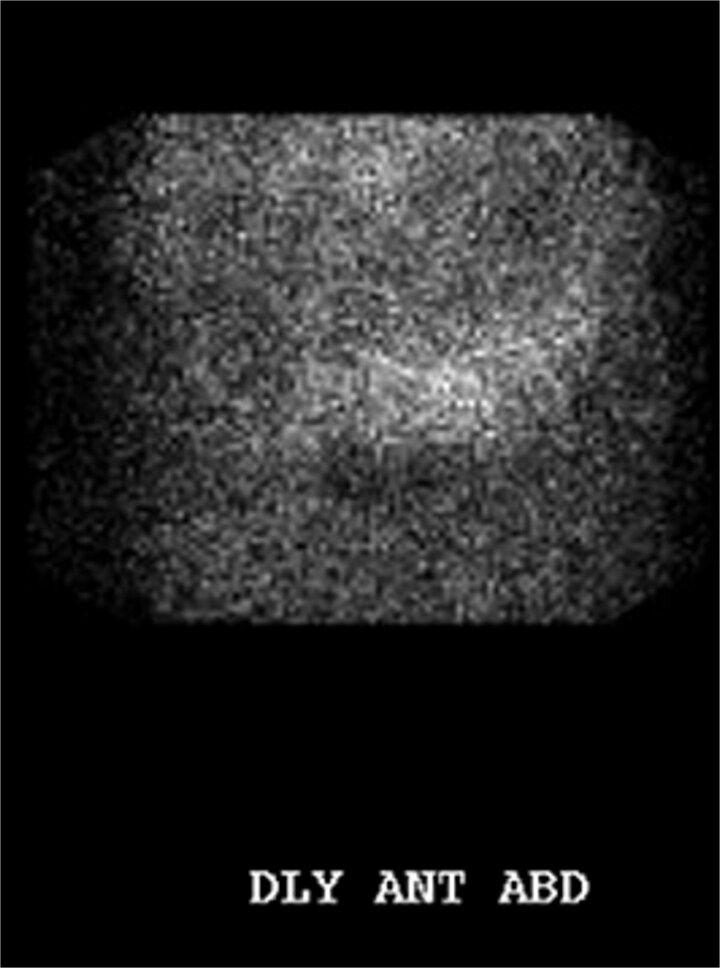
V/Q scan (delayed image) showing tracer seen inside the abdominal cavity, confirming a broncho-abdominal (lung-to-abdomen) fistula.

Alternative etiologies for bronchoperitoneal fistula were considered and ruled out. The patient had no history of thoracic or abdominal trauma, malignancy, chronic infection, or inflammatory disease, and prior imaging did not demonstrate a diaphragmatic defect. Infectious, cardiopulmonary, and intra-abdominal evaluations were unrevealing. Although no diaphragmatic defect was identified on prior imaging or operative reports, it was hypothesized that a subclinical or microscopic diaphragmatic injury related to the prior hiatal hernia repair may have contributed to pneumoperitoneum and PCI, which could have facilitated fistula formation.

The thoracic surgery team was involved in the diagnostic assessment. Although bronchoscopy is often considered in suspected bronchial fistulas, it was deferred due to patient’s clinical stability, absence of pulmonary symptoms and chest imaging being unremarkable, suggesting a low pretest probability of endobronchial pathology. A ventilation-perfusion (V/Q) scan was pursued instead as a functional imaging modality. The detection of radiotracer within the abdominal cavity on delayed imaging suggested transdiaphragmatic passage of gas, consistent with broncho-peritoneal fistula. Although not a standard diagnostic tool for this indication, it provided supportive functional evidence of abnormal communication.

Given the patient’s significant weight loss, malignancy was considered. However, cross-sectional imaging of the chest, abdomen, and pelvis were unremarkable. The patient being clinically stable was discharged with close outpatient follow-up and planned surgical evaluation for fistula repair by thoracic surgery.

## Discussion

Pneumatosis cystoides intestinalis (PCI) is a rare condition that may arise from various underlying mechanisms broadly elucidated by three principal theories: bacterial, mechanical and pulmonary. According to bacterial theory, gas producing bacteria reside or infiltrate the submucosa and generate gas locally. The mechanical theory suggests that increased intrabdominal pressure or mucosal disruption allows gas to traverse into the bowel wall. The pulmonary theory involves rupture of alveoli with air traversing along vascular sheaths into the retroperitoneum and mediastinum, ultimately reaching the bowel wall [9].

In this case, the findings may be supported by a mechanical process involving increased intra-abdominal pressure and cysts rupture contributing to pneumoperitoneum. This could have enabled gas dissection across microscopic diaphragmatic defects leading to development of a broncho-peritoneal fistula. This proposed mechanism aligns best with mechanical theory along with some contribution of pulmonary theory (Fig. 5). Nevertheless, the exact pathophysiology remains uncertain and a definitive causality cannot be established.

**Figure 5:**
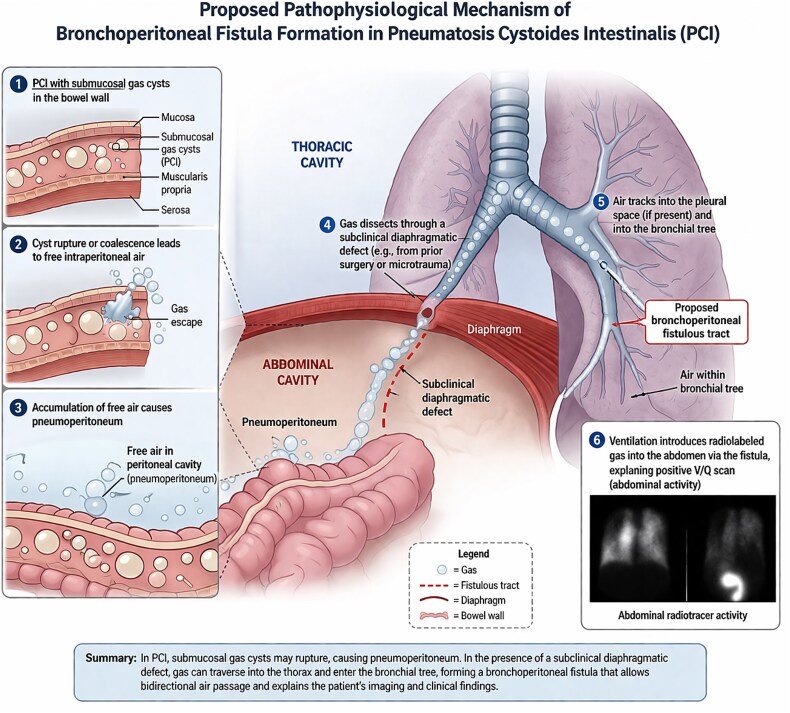
Proposed pathophysiological mechanism of bronco-peritoneal fistula formation in PCI.

While abdominal pain and distension are common in PCI, chronic diarrhea and fecal incontinence, as seen in our patient, especially with significant unintentional weight loss, are rarely emphasized in the literature [10, 11]. These symptoms may result from mucosal disruption, altered intestinal motility, and bacterial overgrowth contributing to impaired absorption.

In our case, the patient presented with chronic symptoms, and imaging revealed pneumoperitoneum, prompting the use of invasive diagnostic modalities to exclude a surgical emergency. This finding supports previous data indicating that PCI is typically a benign condition and does not necessarily require surgical intervention.

Although PCI is generally considered a benign condition, this case was complicated by an extremely rare bronchoperitoneal fistula. Most reported cases described fistula formation secondary to thoracic pathology [3, 4], our case raises the possibility for a reverse mechanism, in which the fistula may have developed in association with PCI which may have been facilitated by an iatrogenic diaphragmatic injury during hiatal hernia repair.

The broncho-peritoneal fistula in this patient may have resulted from pneumoperitoneum secondary to PCI, with gas dissecting through a minor diaphragmatic defect. However, this proposed mechanism remains hypothetical as direct evidence of diaphragmatic disruption or fistula formation pathway was not demonstrated.

This finding may represent a previously unreported complication of PCI that gastroenterologists should be aware of, due to the severe clinical outcomes that can result if it is left unnoticed.

This case also demonstrates a unique and highly unusual pathway for detecting the fistula that was initially suspected due to a halothane-like odor noted intraoperatively, an uncommon clue that prompted a high index of suspicion. Halothane is a volatile anesthetic with sweet odor; similar odors may arise from gases produced in gastrointestinal tract. In this context, halothane-like odor may be a representation of intraluminal gas transmission via an atypical communication. Though nonspecific, this finding may assist as a clinical hint pointing towards an unusual communication. The finding was subsequently confirmed by a V/Q scan in which tracer expected to remain confined to the lungs was detected within the abdominal cavity instead on delayed imaging indicating transdiaphragmatic communication [12].

## Differential diagnosis

The differential diagnosis of this presentation involves various etiologies of pneumoperitoneum such as hollow viscus perforation which was ruled out by absence of peritoneal signs and prior negative exploratory laparotomy. Non-surgical causes including occult diaphragmatic defects and post-surgical changes were also considered; though no defect was identified, a microscopic diaphragmatic injury associated to prior hiatal hernia repair may be possible. Various differentials of V/Q scan findings were also considered including patient contamination, technical artifact, or atypical tracer distribution. Nevertheless, presence of radiotracer within the abdominal cavity along with high clinical suspicion supported the diagnosis of a broncho-peritoneal fistula. Chronic infection, malignancy or malabsorption were also considered as the patient experienced fecal incontinence, diarrhea and weight loss but imaging and endoscopy were unremarkable, making PCI the most likely cause of these symptoms.

## Limitations

This case has multiple limitations, the main one being lack of definitive anatomical confirmation of the broncho-peritoneal fistula since diagnosis was made based on functional imaging rather than direct visualization. In addition, a causal relation between PCI and fistula formation cannot be conclusively established, making the proposed pathophysiological association hypothetical.

## Conclusion

This case describes a rare and diagnostically challenging presentation of bronchoperitoneal fistula occurring in the setting of PCI and pneumoperitoneum. While a causal relationship cannot be confirmed, the case highlights a potential association that warrants awareness and further investigation.

## Notes

Clinical Key Message: This case describes a rare bronchoperitoneal fistula complicating pneumatosis cystoides intestinalis (PCI), suggesting an atypical, non-thoracic origin. It highlights PCI as a potential source for fistulization to the bronchial tree, a critical diagnostic consideration in patients with pneumoperitoneum and respiratory symptoms.
